# A case–control study of the clinical and economic impact of infections caused by Carbapenemase-producing Enterobacterales (CPE)

**DOI:** 10.1007/s15010-024-02268-z

**Published:** 2024-05-03

**Authors:** Inmaculada López Montesinos, Aina Carot-Coll, Maria Milagro Montero, Luisa Sorli Redó, Ana Siverio-Parès, Sandra Esteban-Cucó, Xavier Durán, Silvia Gomez-Zorrilla, Juan Pablo Horcajada

**Affiliations:** 1 Infectious Disease Service, Hospital del Mar, Institut Hospital del Mar d’Investigacions Mèdiques (IMIM), Passeig Marítim de La Barceloneta, 25-29, 08003 Barcelona, Spain; 2 Universitat Pompeu Fabra (UPF), Barcelona, Spain; 3 Microbiology Service, Laboratori de Referència de Catalunya, El Prat de Llobregat (Barcelona), Spain; 4 Methodology and Biostatistics Support Unit, Institut Hospital del Mar d’Investigacions Mèdiques (IMIM), Barcelona, Spain; 5 CIBER de Enfermedades Infecciosas, Instituto de Salud Carlos III (CIBERINFEC ISCIII), Madrid, Spain

**Keywords:** Carbapenem-Resistant Enterobacterales, Carbapenemase, Multiple drug resistance, Mortality, Hospital costs

## Abstract

**Purpose:**

The aim was to analyse the clinical and economic impact of carbapenemase-producing Enterobacterales (CPE) infections.

**Methods:**

Case–control study. Adult patients with CPE infections were considered cases, while those with non-CPE infections were controls. Matching criteria were age (± 5 years), sex, source of infection and microorganism (ratio 1:2). Primary outcome was 30-day mortality. Secondary outcomes were 90-day mortality, clinical failure, hospitalisation costs and resource consumption.

**Results:**

246 patients (82 cases and 164 controls) were included. *Klebsiella pneumoniae* OXA-48 was the most common microorganism causing CPE infections. CPE cases had more prior comorbidities (p = 0.007), septic shock (p = 0.003), and were more likely to receive inappropriate empirical and definitive antibiotic treatment (both p < 0.001). Multivariate analysis identified septic shock and inappropriate empirical treatment as independent predictors for 7-day and end-of-treatment clinical failure, whereas Charlson Index and septic shock were associated with 30- and 90-day mortality. CPE infection was independently associated with early clinical failure (OR 2.18, 95% CI, 1.03–4.59), but not with end-of-treatment clinical failure or 30- or 90-day mortality. In terms of resource consumption, hospitalisation costs for CPE were double those of the non-CPE group. CPE cases had longer hospital stay (p < 0.001), required more long-term care facilities (p < 0.001) and outpatient parenteral antibiotic therapy (p = 0.007).

**Conclusions:**

The CPE group was associated with worse clinical outcomes, but this was mainly due to a higher comorbidity burden, more severe illness, and more frequent inappropriate antibiotic treatment rather than resistance patterns as such. However, the CPE group consumed more healthcare resources and incurred higher costs.

**Supplementary Information:**

The online version contains supplementary material available at 10.1007/s15010-024-02268-z.

## Introduction

The emergence and spread of multidrug-resistant (MDR) bacterial infections has become a major public health concern, as these bacteria are prone to human-to-human transmission and are difficult to treat [1]. They have traditionally been linked to healthcare settings [2], where they can be responsible for outbreaks among patients, but the problem is further exacerbated by reports in recent years of an increase in community settings [3]. In relation to the healthcare sector, the spread of MDR bacteria is expected to cause over 10 million deaths per year by 2050, and in terms of global economic output, production losses are estimated at billions of dollars [4].

In recent years, enzyme-producing Enterobacterales that confer resistance to beta-lactam antibiotics have appeared and spread all around the world [1, 4, 5]. This includes carbapenemase-producing Enterobacterales (CPE), resistant to carbapenem antibiotics, the traditional cornerstone for the treatment of severe infections caused by gram-negative bacteria [4, 5].

The 2022 European Centers for Disease Prevention and Control (ECDC) annual epidemiological report showed that 10.9% of *Klebsiella pneumoniae* isolates were carbapenem-resistant, with an almost 50% increase between 2019 and 2022 [6]. Not surprisingly, the World Health Organization (WHO) has declared antibiotic resistance as one of the 10 public health threats facing humanity, and CPE as one of the critical pathogens for which new antibiotics are urgently needed [7, 8].

Infections caused by CPE are difficult to treat because there are few options available due to their extensive resistance profile. Until quite recently, patients with CPE infections were treated with polymyxins or aminoglycosides in monotherapy or in combination with other antibiotics with suboptimal clinical results and high rates of toxicity [9, 10] With the development of novel drugs during the last years (such as ceftazidime-aztreonam, imipenem-relebactam, meropenem-vaborbactam, or cefiderocol) more antibiotic options are available for the management of CPE infections. These new drugs should be reserved for treatment of MDR gram negative bacteria with limited or no alternative options, with antimicrobial stewardship programs to limit the development of resistance to the novel antibiotic agents [11]. Despite the development of these novel antibiotics, treatment options for *metallo beta-lactamase* (MBL) mediated resistance patterns are limited. Although previous studies [10, 12–17] have analysed the clinical and economic burden related to CPE infections, in the present work we assessed clinical response, mortality, resource consumption and economic impact as a whole to give a more comprehensive view of a global problem. In the present study, we hypothesised that patients admitted with active infections caused by CPE have worse clinical outcomes, consume more resources and incur higher economic costs compared to patients admitted with infections caused by susceptible or non-MDR bacteria. The aim of this study was to analyse the clinical and economic impact of CPE infections.

## Patients and methods

### Study design

This was a retrospective case–control study of patients with active infections caused by CPE and non-MDR bacteria between January 2010 and October 2022. The research was carried out at the Parc de Salut Mar, in Barcelona (Spain).

During this time, every positive culture from a clinical sample showing growth of CPE isolates was examined; only those patients with criteria for infection were included as cases. Patients with a positive culture but no signs of infection were considered as colonised and excluded. Inclusion criteria were age 18 years or older, isolation of CPE in a clinical sample, and signs and symptoms of infection in the same focus as the culture. Outpatients were only included if they required hospitalisation due to the infection. Only the first episode of infection per patient was considered. Exclusion criteria were: (i) polymicrobial infections, (ii) colonisation without active infection, and (iii) end-of-life patients (palliative care patients) who did not receive active therapy for the infection.

The control group was made up of patients with active non-MDR bacterial infections. The matching criteria were based on controls and cases sharing a susceptible strain of the same bacterial species, focus of infection, and same sex and age ± 5 years.

As the initial control groups did not reach the sample size required for statistical analysis when all four matching criteria were combined (see further), the requirement for control inclusion was relaxed to both the same focus of infection and susceptible strain of the same microorganism. Matching by age and/or sex was applied whenever possible.

Sample size determination was based on the results of a previous study [18] to detect a 10% difference in 30-day mortality between CEP and non-MDR bacteria; statistical power was set at 80% and alpha error at 0.05. The sample size ratio for matching was 1:2 and 82 cases and 164 controls were obtained.

Patients were followed for 90 days from the onset of the infection. The follow-up was carried out through the electronical clinical records, which integrates hospital, primary care, homecare, long-term facilities, and social services records in the healthcare region of the hospital setting.

### Outcomes

The primary outcome variable was all-cause mortality at 30 days, considering day 1 as the first day of positive culture. Secondary outcomes were clinical failure at day 7 (early clinical failure) and end of treatment, crude mortality at day 90, hospitalisation costs and resource consumption.

### Data collection, variables, and definitions

Information on the study variables was obtained from an electronic medical record and economic data through the hospital’s economic database.

The variables considered for the clinical impact study were age, gender, underlying diseases according to the Charlson comorbidity index [19], antibiotic treatment in the previous 3 months, hospitalisation, invasive devices (urinary catheter, vascular access) or dialysis, microorganism isolated, source of infection, empirical and definitive treatment, duration, and antibiotic-related side effects.

An assessment of clinical severity was made for septic shock and intensive care unit (ICU) admission in the first 72 h, as well as a Sequential Organ Failure Assessment (SOFA) [20]. Septic shock was defined according to the Sepsis-3 definition [21]. In case of bacteraemia, the Pitt score [22] was applied.

Clinical failure was defined as persistence or worsening of signs and/or symptoms of the infection and/or death. Empirical antibiotic therapy was considered appropriate when at least one antibiotic active in vitro active antibiotic was given against the microorganism was administered.

For the consumption of healthcare resources, several variables were considered: length of stay, need for outpatient parenteral antibiotic treatment and long-term care facilities, and readmissions at 90 days of follow-up. Finally, the overall cost of hospitalisation, pharmacy and diagnostic tests for each patient were also assessed.

### Microbiological data

MDR was defined as resistance to at least one agent in three or more antimicrobial categories for each organism, according to current standard definitions [23]. Non-MDR was considered when resistance to fewer than three active antimicrobial categories was observed. According to this classification, the antimicrobial categories used to define MDR in Enterobacteriales were: aminoglycosides, anti-MRSA cephalosporins, antipseudomonal penicillins + *β*-lactamase inhibitors, carbapenems, 1st and 2nd generation cephalosporins, 3rd and 4th generation cephalosporins, cephamycins, fluoroquionolones, folate pathway inhibitors (trimethoprim-sulphamethoxazole), glycylcyclines (tigecycline), monobactams, penicillins + *β*-lactamase inhibitors, phenicols (chloramphenicol), phosphonic acids, polymyxins and tetracyclines [19].

Routine identification and susceptibility testing of causative microorganisms was performed using automated systems (the Vitek-2® [BioMérieux] for blood cultures, and the MicroScan® WalkAway [Beckman-Coulter] for other sample types). Results were interpreted according to the European Committee on Antimicrobial Susceptibility Testing (EUCAST) standards in force at the time of culture. Carbapenemases were identified by multiplex PCR assay, using the LightMix® Modular Carbapenemase panel (Roche Diagnostics).

### Statistical analysis

Qualitative variables were shown as numbers and percentages, and quantitative variables as median and interquartile range (IQR). Quantitative variables were analysed using the Student’s t-test or Mann–Whitney U test, and qualitative variables using the Chi-Square (χ2) or Fisher’s exact test, as appropriate.

A logistic regression model was used to examine variables associated with clinical failure at day 7 and at end of treatment, expressing the results as odds ratio (OR) and 95% confidence interval (CI).

Kaplan–Meier survival curves and the log-rank test were used to detect differences in 30- and 90-day mortality.

The Cox proportional hazards model was used to perform multivariate survival analyses for 30- and 90-day mortality. Results were expressed as hazard ratio (HR) and 95% CI. The proportional hazards assumption was tested.

To control for confounding, variables in the crude analysis that were associated with exposure (p ≤ 0.20) or outcome (OR or HR < 0.65 or > 1.5), and those that were not considered to be intermediate variables between exposure and outcome were candidates for multivariate analysis. Variables leading to substantial confounding if removed (10% or more change in the coefficient estimate) were retained in the model, along with exposure (CPE group). Manually selected, backward stepwise regression was applied. Variables with > 25% missing values were not considered in multivariate analysis.

For the economic analysis, median regression (to deal with non normal dependent variables) was used. Results were expressed as the difference in medians (DM). Interpretation of the coefficients was based on the difference of means, as it was for the interpretation of coefficients in multiple linear regression.

All p-values were 2-tailed and statistical significance was set at ≤ 0.05. Statistical analyses were performed using STATA 15.1. STROBE guidelines were used for the reporting of the study (Supplementary Table S1).

### Ethics

The Clinical Research Ethical Committee of the Parc Salut Mar approved this study (registration no. 2022/1046). Written informed consent was waived due to its observational and retrospective nature.

## Results

**Fig. 1:**
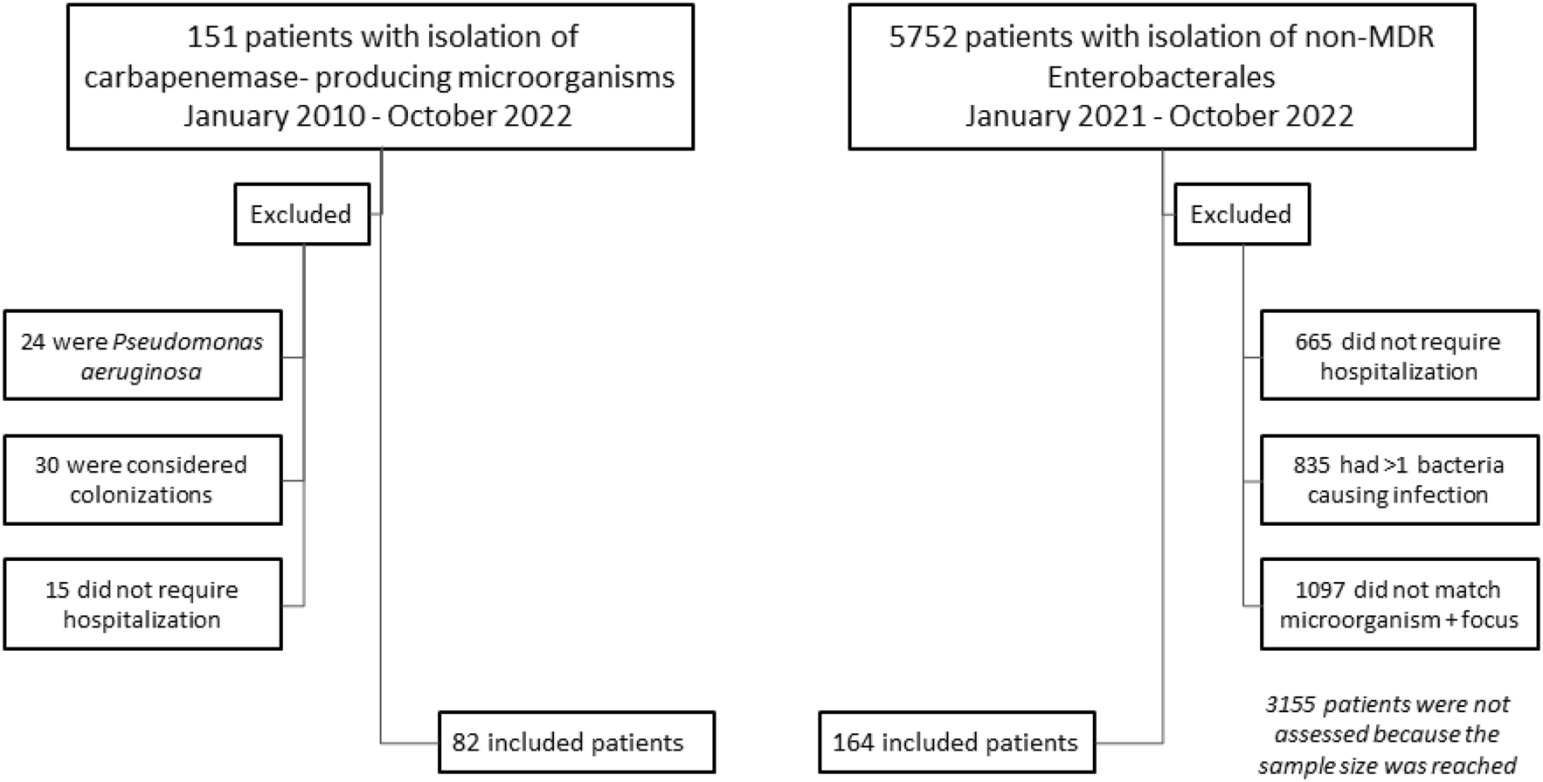
Flowchart of patients included in the study. Abbreviations: MDR (MDR, multidrug-resistant)

A total of 246 patients eligible for inclusion in the study were studied: 82 infections were caused by CPE (cases) and 164 by non-MDR Enterobacterales (controls) (Fig. 1).

Of the CPE isolates, 58 (70.73%) were oxacillin-type carbapenemases (OXA-48); 16 (19.51%) were Verona integron–encoded metallo-β-lactamase (VIM), and 4 (4.88%) each were *Klebsiella pneumoniae* carbapenemase (KPC) and New Delhi metallo-β-lactamase (NMD). In terms of antimicrobial susceptibility, all CPE isolates were susceptible to colistin. Of the OXA-48 tested: 22/23 (95.7%) were susceptible to ceftazidime-avibactam, 35/49 (71.4%) to amikacin, 18/28 (64.3%) to fosfomycin, 18/57 (31.6%) to trimethoprim-sulfamethoxazole and 7/57 (12.3%) to ciprofloxacin. Susceptibility rates were lower for VIM isolates: 0/3 (0%) to ceftazidime-avibactam, 8/14 (57.1%) to amikacin, 1/15 (6.7%) to trimethoprim-sulfamethoxazole, 1/16 (6.3%) to ciprofloxacin, (1/16, 6.3%), except against fosfomycin (7/8, 87.5%). All KPC and NMD strains were resistant to ciprofloxacin. Susceptibility to fosfomycin was found in 66.7%,of NDM, and to trimethoprim-sulfamethoxazole in 25% of NDM and KPC.

### Demographic and epidemiological information, clinical features, and therapeutic management

Differences in baseline characteristics, severity assessments and therapeutic management are shown in Table 1. Considering the matching criteria (age, sex, microorganism, and site of infection) there were no differences among between the studied.

**Table 1:** Univariate analysis of patient characteristics for non-MDR (controls) and CEP (cases) infections

Clinical variable	Non-MDR (n = 164) No. %	CPE (n = 82) No. %	p-value
*Demographic and epidemiological information*			
Age, m (IQR); years	74 (59–84)	73 (61–80.5)	0.7821
Male sex	88 (53.66)	54 (65.85)	0.068
Charlson Index, m (IQR)	3 (1–5)	4 (2–6)	**0.007**
Ischaemic heart disease	35 (21.3)	13 (15.9)	0.306
Heart insufficiency	32 (19.5)	20 (24.4)	0.377
Peripheral vascular disease	36 (22)	17 (20.7)	0.826
Cerebrovascular disease	21 (12.8)	15 (18.3)	0.251
Dementia	10 (6.1)	17 (20.7)	** < 0.001**
Chronic lung disease	19 (11.6)	21 (25.6)	**0.005**
Connective tissue disease	13 (7.9)	1 (1.2)	**0.039**
Ulcerative disease	11 (6.7)	7 (8.5)	0.604
Mild liver disease	9 (5.5)	6 (7.3)	0.572
Moderate-severe liver disease	11 (6.7)	13 (15.9)	**0.023**
Diabetes: No organic damage	38 (23.2)	14 (17.1)	0.270
Diabetes: Organic damage	26 (15.9)	14 (17.1)	0.807
Hemiplegia	0 (0)	3 (3.7)	**0.036**
Renal failure	37 (22.6)	38 (46.3)	** < 0.001**
Solid tumour	35 (21.3)	16 (19.5)	0.739
Metastatic tumour	17 (10.4)	6 (7.3)	0.439
Leukaemia	1 (0.6)	2 (2.4)	0.258
Malignant lymphoma	3 (1.8)	0 (0)	0.553
Immunosuppression	13 (7.9)	5 (6.1)	0.604
*HCA risk factors*			
Prior hospital stays (3 months)	74 (45.1)	56 (68.3)	** < 0.001**
Prior ATB treatment (3 months)	48 (29.3)	68 (82.9)	** < 0.001**
Medical devices (3 months)	81 (49.4)	67 (81.7)	** < 0.001**
Urinary catheter	46 (28)	46 (56.1)	** < 0.001**
Vascular access	68 (41.5)	61 (74.4)	** < 0.001**
Dialysis	4 (2.4)	5 (6.1)	0.165
Hospital acquired infection	62 (37.8)	42 (51.2)	**0.045**
Previous colonisation	10 (6.1)	21 (25.6)	** < 0.001**
*Severity assessment and management*			
Septic shock	11 (6.71)	15 (18.3)	**0.005**
ICU stay (first 72 h)	14 (8.5)	16 (19.5)	**0.013**
SOFA ≥ 1	14 (8.54) (14/164)	17 (30.9) (17/55)	** < 0.001**
Pitt Score ≥ 1	11 (6.7) (11/164)	17 (31.5) (17/54)	** < 0.001**
*Therapeutic management*			
Appropriate empirical treatment	113 (68.9)	7 (8.64)	** < 0.001**
Appropriate definitive treatment	142 (95.30)	66 (80.49)	** < 0.001**
Duration of treatment, m(IQR); days	11 (8–15)	15 (11–24)	**0.0002**
Antibiotic-related side effects	0 (0)	9 (10.98)	** < 0.001**

A *p* value was considered statistical significant at ≤ 0.005

Data are presented as n (%), unless otherwise specified. Abbreviations: *m* (median), *IQR* (interquartile range), *MDR* (MDR (multidrug-resistant), *CPE* (carbapenemase-producing Enterobacteriaceae), (*HCA* (healthcare-associated), *ATB* (antibiotic), *ICU* (intensive care unit), *SOFA* (Sequential Organ Failure Assessment)

**Fig. 2:**
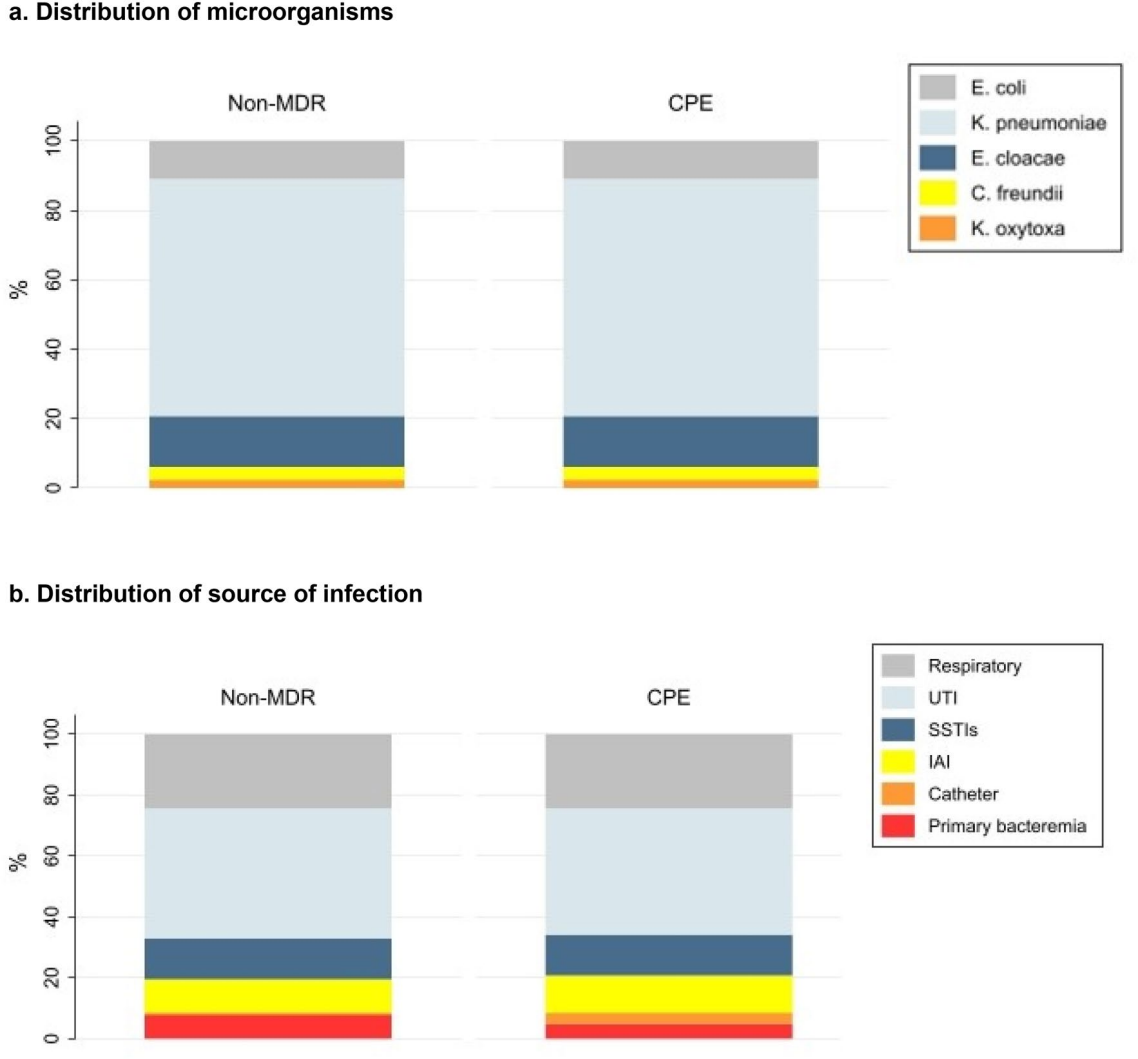
Distribution of microorganisms (**A**) and sources of infection (**B**). *Distribution of microorganisms (non-MDR vs CPE group): *Escherichia. coli* 18 (11%) vs 9 (11%); *Klebsiella pneumoniae* 112 (68.3%) vs 56 (68.3%); *Enterobacter cloacae* 24 (14.6%) vs 12 (14.6%); *Citrobacter freundii* 6 (3.7%) vs 3 (3.7%) and *Klebsiella oxytoca* 4 (2.4) vs 2 (2.4%). All p values = 1. Abbreviations: *MDR* (MDR (multidrug-resistant), *CPE* (carbapenemase-producing Enterobacterales). *Distribution of source of infection (non-MDR vs CPE group): Respiratory 40 (24.4%) vs 20 (24.4%), p = 1; UTI 70 (42.7%) vs 34 (41.5%), p = 0.855; SSTIs 22 (13.4%) vs 11 (13.4%), p = 1; Catheter 1 (0.6%) vs 3 (3.7%), p = 0.109; IAI 18 (11%) vs 10 (12.2), p = 0.776 and primary bacteraemia 13 (7.9) vs 4 (4.9%), p = 0.374. Abbreviations: *MDR* (MDR (multidrug-resistant), *CPE* (carbapenemase-producing Enterobacterales), *UTI* (Urinary Tract Infection, includes pyelonephritis, prostatitis and urosepsis), *SSTIs* (Skin and Soft Tissue Infections, includes cellulitis, abscess, myositis and fasciitis), *IAI* (Intra-abdominal Infections, includes *Clostridioidesum difficile Clostridioides difficile*, gastrointestinal tract infection and necrotising enterocolitis)

The most frequently isolated microorganisms and sites of infection can be seen in Fig. 2(A/B). Overall, 20% of patients had bloodstream infections: 30 (18.3%) in the non-MDR group and 20 (24.4%) in the CPE cases (p = 0.263).

CPE patients had more underlying diseases as assessed by the Charlson comorbidity index than those with non-MDR bacteria (median points, 4 [IQR 2–6] vs. 3 [IQR 1 to 5]; p = 0.007). Dementia, lung, kidney and liver diseases were seen more in the CPE group.

CPE cases were more related to healthcare activity [previous hospital stays (p < 0.001), previous antibiotic exposure (p < 0.001) and previous use of invasive devices (p < 0.001) in the previous 3 months; had more hospital-acquired infections (p = 0.045) and were more likely to be previously colonised by an MDR microorganism (p < 0.001).

At the onset of infection, CPE patients were more seriously ill, reflected in a higher proportion of septic shock (p = 0.003), ICU stay (p = 0.008), and higher SOFA and Pitt scores (p < 0.001 in both cases).

CPE cases were more likely to receive inappropriate antibiotic treatment (p < 0.001), have longer courses of antibiotic therapy (p < 0.001) and more antibiotic-related side effects (p < 0.001). Most common target antibiotic regimens used for CPE infections included: ceftazidime-aztreonam in monotherapy (18 patients) or in combination with aminoglycosides or polymyxins (4 patients); tigecycline in monotherapy (3 patients) or tigecycline in combination with carbapenems, polymyxins, aminoglycosides and/or fluoroquinolones (10 patients); colistin in monotherapy (3 patients) or in combination with carbapenems, fosfomycin or others (6 patients) and fosfomycin in monotherapy (5 patients).

### Clinical outcomes

CPE infections had higher rates of clinical failure at day 7 (79.3 vs. 39%; p < 0.001) and at the end of treatment (20.7% vs. 4.3%; p < 0.001), as well as higher crude mortality at 90 days (25.6% vs. 14.6% p = 0.036). However, no differences were observed for 30-day mortality (11% vs. 6.1%, p = 0.117). Higher 30-day mortality rates were observed for CPE cases with pneumonia or bacteraemia (20%) or septic shock (40%). The microbiological clearance rate at day 90 was higher in non-MDR infections (n = 69/79; 90.8 vs. n = 32/45; 71.1%; p = 0.005).

Table 2 shows the multivariate analysis of clinical failure at day 7 and at the end of treatment. CPE infection was independently associated with clinical failure at day 7 (adjusted OR 2.18; CI 1.03–4.59; p = 0.041) but not at the end of treatment (Table 2).

**Table 2:** Crude and adjusted measures of association between different variables and clinical failure at day 7 and at end of treatment

Day 7	Crude OR (95%CI)	p-value	aOR (95%CI)	p-value
Charlson Index	1.09 (0.99–1.21)	0.062	1.05 (0.93–1.17)	0.430
Septic shock	5.81 (1.94–17.41)	**0.002**	4.51 (1.35–15.03)	**0.014**
Appropriate empirical treatment	0.13 (0.07–0.23)	** < 0.001**	0.2 (0.1–0.39)	** < 0.001**
CPE infection	5.97 (3.22–11.1)	** < 0.001**	2.18 (1.03–4.59)	**0.041**
*End of treatment*				
Charlson Index	1.22 (1.05–1.41)	**0.010**	1.17 (1–1.38)	0.057
Septic shock	9.20 (3.53–23.97)	** < 0.001**	7.35 (2.5–21.49)	** < 0.001**
Appropriate empirical treatment	0.04 (0.01–0.28)	**0.001**	0.07 (0.01–0.58)	**0.014**
CPE infection	5.87 (2.33–14.81)	** < 0.001**	1.7 (0.59–4.94)	0.329

A *p* value was considered statistical significant at ≤ 0.005

Abbreviations: *OR* (Odds ratio), *aOR* (adjusted odds ratio), *CI* (Confidence Interval), *CPE* (Carbapenemase-producing Enterobacterales)

**Fig. 3:**
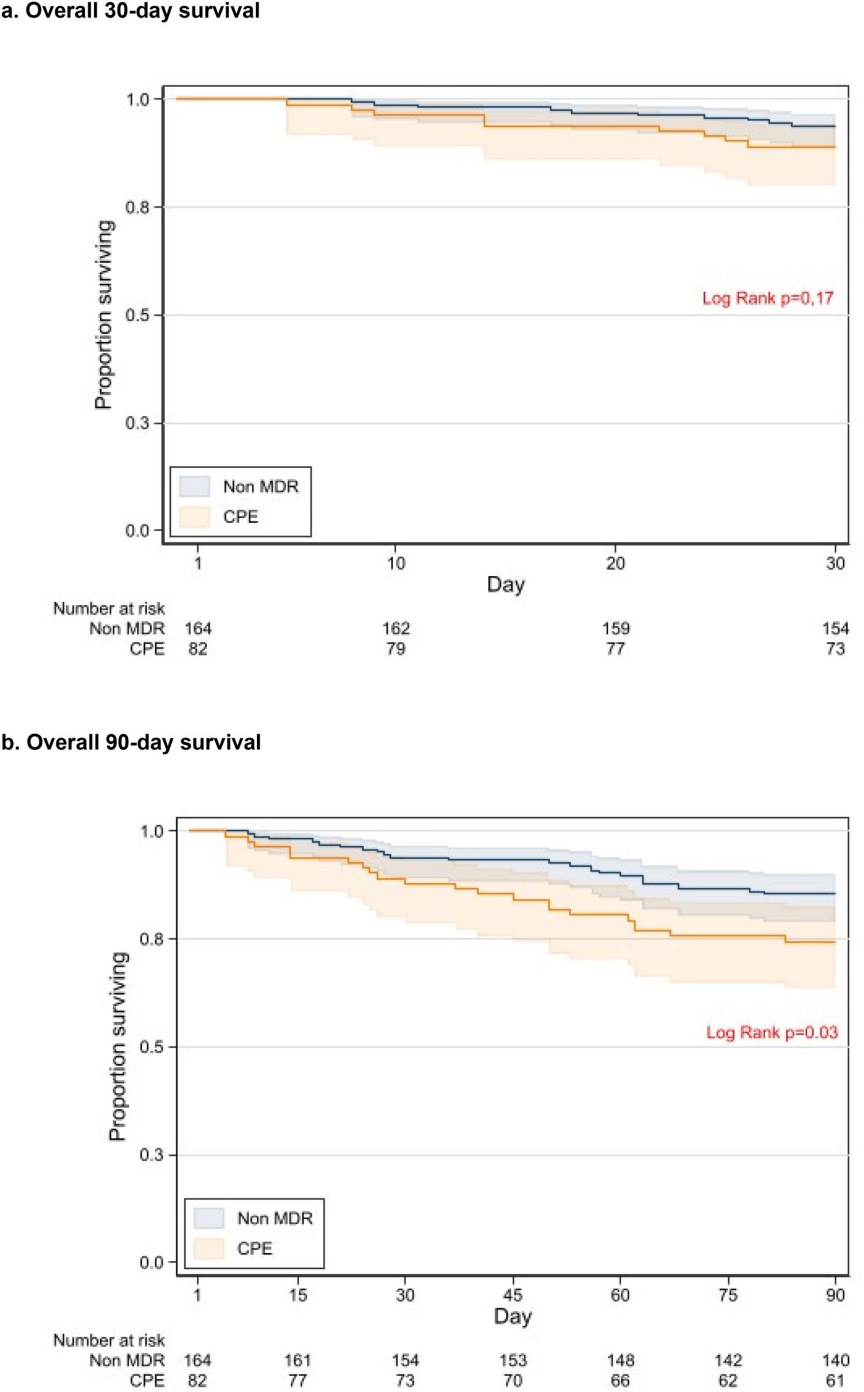
Cumulative Kaplan–Meier estimates in the non-MDR and CPE groups for (**A**) the probability of overall survival at day 30, and (**B**) at day 90

Figure 3a and b show the respective Kaplan–Meier curves for 30- and 90-day mortality by groups. After adjusting for confounders, CPE infection was not associated with either 30-day or 90-day mortality (Table 3).

**Table 3:** Crude and adjusted measures of association between different variables and 30- and 90-day mortality

30-day mortality	Crude HR (95%CI)	p-value	aHR (95%CI)	p-value
Charlson Index	1.19 (1.03–1.37)	0.020	1.19 (1.02–1.39)	**0.028**
Septic shock	11.51 (4.67–28.4)	< 0.001	11.31 (4.4–29.1)	** < 0.001**
Appropriate empirical treatment	0.26 (0.09–0.80)	0.019	0.39 (0.11–1.36)	0.137
CPE infection	1.86 (0.76–4.58)	0.177	0.65 (0.24–1.76)	0.392
*90-day mortality*				
Charlson Index	1.29 (1.18–1.42)	< 0.001	1.29 (1.17–1.43)	** < 0.001**
Septic shock	4.93 (2.58–9.41)	< 0.001	5.16 (2.60–10.23)	** < 0.001**
Appropriate empirical treatment	0.38 (0.19–0.72)	0.003	0.56 (0.26–1.20)	0.138
CPE infection	1.90 (1.06–3.41)	0.032	0.86 (0.43–1.72)	0.673

A *p* value was considered statistical significant at ≤ 0.005

Abbreviations: *HR* (hazard ratio), *aHR* (adjusted hazard ratio), *CI* (Confidence Interval), *CPE* (Carbapenemase-producing Enterobacterales)

### Resource consumption and costs

Compared with the non-MDR group, patients with CPE infection had longer hospital stays (median days, 35.5 [IQR 16–60] vs. 18 [10–38.5]; p < 0.001), more frequently needed long-term care facilities (p < 0.001) and outpatient parenteral antibiotic therapy (p = 0.007).

With respect to the economic burden, the costs of hospitalisation, pharmacy, and diagnostic tests were all higher in CPE patients (all p < 0.001). Hospitalisation costs for CPE patients were more than double those associated with non-MDR cases, with a median difference of 13,417.38 € between groups (p < 0.001) (Table 4). These results were also confirmed after adjusting for confounders (Table 5).

**Table 4:** Healthcare resource consumption and costs of hospital stay

	non-MDR (n = 164)	CPE (n = 82)	p-value
*Healthcare resource consumption*			
Length of stay, days	18 (10–38.5)	35.5 (16–60)	** < 0.001**
Need for OPAT, n (%)	2 (1.23)	5 (8.33)	**0.007**
Need for long-term care facilities	39 (24.07)	33 (55)	** < 0.001**
Readmissions (3 months)	65 (40.12)	31 (52.54)	0.123
*Costs, €*			
Overall cost of hospitalisation	13,007.56 (6320.30- 28,584.56)	26,424.94 (14,190.52–46,541.73)	** < 0.001**
Pharmacy	422.46 (162.58–2020.835)	2360.99 (780.34–6251.47)	** < 0.001**
Diagnostic tests	962.78 (414.94–3163.58)	1707.23 (520.88–3778.88)	** < 0.001**

A *p* value was considered statistical significant at ≤ 0.005

Data are presented as median (m) and interquartile range (IQR) unless otherwise specified. Cost values are presented in Euros (€). Abbreviations: *MDR* (multidrug-resistant), *CPE* (carbapenemase-producing Enterobacterales), *OPAT* (outpatient parenteral antibiotic therapy)

**Table 5:** Univariate and multivariate analyses of overall costs of hospitalisation

	DM (95%CI)	p-value	Adjusted (95%CI)	p-value
*Hospital stay costs*				
Charlson index	– 545.28 (– 1849.06 to 758.49)	0.411	– 871.52 (– 2000.71–257.66)	0.130
Septic shock	17,286.74 (7168.13–27,405.35)	**0.001**	14,924.63 (5079.79–24,769.47)	**0.003**
Appropriate empirical treatment	– 5982.69 (– 12,826.67 to 861.28)	0.086	163.55 (– 7146.86 to 7473.96)	0.965
CPE infection	13,386.73 (6669.18–20,104.28)	** < 0.001**	9097.61 (1131.87–17,063.34)	**0.025**

A *p* value was considered statistical significant at ≤ 0.005

Abbreviations: *DM* (Difference in Medians), *CI* (Confidence Interval), *CPE* (carbapenemase-producing Enterobacterales)

## Discussion

Following the WHO alert that CPE are becoming a major public health concern [7, 8], more studies are being conducted to gain a better understanding of this type of infection and to evaluate its impact. This case–control study evaluated the burden of CPE infections on clinical and economic outcomes.

CPE infections did not result in increased mortality at 30 or 90 days, or in higher clinical failure rates at the end of antibiotic treatment. On the other hand, CPE cases were independently associated with higher early clinical failure.

Conflicting results have been published on the impact of CPE infections on clinical outcomes [12–15]. Apart from carbapenem resistance itself, other factors, such as patient comorbidities, host response, source and severity of the infection, therapeutic management, type of microorganism, mechanism of carbapenem resistance and virulence factors, are likely to play a role in these discrepancies.

In our cohort, the overall 30-day mortality in CPE cases was 11%, which is a lower proportion than has been previously reported [13, 15, 24, 25]. However, mortality was higher in patients with pneumonia or bacteraemia (20%) and in those with septic shock (40%). In addition, more than 40% of included patients had a urinary source, with a mortality rate of 6% in this subgroup.

Baseline comorbidities and septic shock at the infection were found to be predictors of 30- and 90-day mortality, as previously reported [12–15, 25]. Although inappropriate antibiotic therapy was not independently associated with higher mortality, it was independently associated with increased clinical failure. CPE cases were three times more likely than non-MDR cases to receive inappropriate empiric and definitive antibiotic therapy (91.4% vs. 31.1% and 19.5% vs 4.5%, respectively; both p < 0.001). Given that the presence of CPE substantially increases the risk of receiving inappropriate antibiotic therapy and that this single factor is relatively modifiable, active surveillance and use of local data should be applied to guide treatment decisions and improve outcomes in CPE infections.

In terms of resource consumption, patients with CPE infection required longer hospital stays and more frequently used long-term care facilities and outpatient parenteral antibiotic therapy, which resulted in significantly higher costs for hospitalisation, pharmacy, and diagnostic tests. These findings are consistent with previous reports [12, 26–29]. Furthermore, CPE cases were more likely to receive longer courses of antibiotics and to have more antibiotic-related side effects. Following on from this, due to the susceptibility profile of CPE infections, there were few, if any, opportunities to use oral antibiotic treatment in our cohort, which perpetuates the vicious cycle of longer length of stay or need for outpatient parenteral antibiotic therapy and translates into higher costs, both of which could be avoided in non-MDR cases.

In general, a patient with a CPE infection should be considered as more complex, vulnerable, and costly. It should raise alarms in the healthcare system, since these patients have more comorbidities and severe illnesses, which involves a greater economic burden and special measures (such as specific environmental cleanup cleaning activities of the environment, isolation rooms and consumables, faecal and medical waste management, staff education). Infection control measures are urgently needed to reduce the selection and spread of these bacteria.

This study has some limitations. First, data were collected retrospectively from medical charts, which makes the results susceptible to selection and reporting biases. We cannot exclude the possibility that there were residual confounding factors not taken into account that could have influenced the results. Second, a single hospital centre does not represent all CPE infections. In our study, *K. pneumoniae* OXA-48 was the most frequent causative microorganism of CPE infections, which is consistent with epidemiological studies in Spain [30, 31], but these results are not transferable to settings with a different epidemiology. Third, the sample size precluded analysis of certain subgroups, such as bacterial genus, species and carbapenemase type or the source of infection. Fourth, although considerable efforts were made to adjust for four matching criteria, not all included patients were fully matched. However, no statistically significant differences in matching characteristics were observed between groups. Fifth, our study was conducted between 2010 and 2022, and therefore, during the first years of the study novel β-lactam/β-lactamase inhibitor combinations or cefiderocol were not available. This could have conditioned a worse outcome due to the use of alternative treatments (such as polymyxins), less effective and with more adverse events. However, as the access to new antibiotics is not homogenous, we consider that our results are also of interest since in some settings patients are still managed with antibiotic combinations of “old drugs”. Finally, the inclusion of nosocomial CPE infections may have overestimated the total costs of hospitalisation in this group. The estimation of costs attributable to these infections is methodologically challenging since they may be biased by the initial reason for admission. After using multivariate analysis to reduce this bias, the CPE cases were still more costly.

Our study has several strengths. First, only infected patients were included, unlike other cohorts in which colonised cases were also taken into account [14]. In keeping with this, about 20% of patients in each group had a bloodstream infection or septic shock, which is a good indicator that the patients were appropriately selected. Second, only CPE were assessed, and not carbapenem-resistant isolates, in which underlying resistance mechanisms other than carbapenemase production may have been involved. Third, several outcomes were considered (clinical failure, mortality, resource consumption, and economic burden), to give a more comprehensive view of this problem. Finally, the inclusion of a heterogeneous sample of CPE reflects the day-to-day reality of hospitals.

In conclusion, this study found that patients with CPE infections had worse clinical outcomes, although this was explained by higher patient comorbidity, more frequent inappropriate empirical treatment, and more severe infection at the onset of infection, not by antibiotic resistance per se. Nevertheless, CPE infections consumed more healthcare resources and the overall costs of hospitalisation were higher.

## Key points

- Carbapenemase-producing Enterobacterales (CPE) infections are increasing worldwide.
- The clinical and economic burden of CPE infections was evaluated.
- CPE was related to sicker patients, inappropriate treatment and early clinical failure.
- CPE cases had longer hospital stay and higher costs.
- CPE could be a marker of more complicated and costly admissions.

## Supplementary Information

Below is the link to the electronic supplementary material.

Supplementary file1 (DOCX 18 KB)

## Data Availability

Data will be made available upon personal contact to authors.
