# Supplementary material for: A case–control study of the clinical and economic impact of infections caused by Carbapenemase-producing Enterobacterales (CPE)
Source: Infection. 2024 May 3;52(6):2241–52. doi: 10.1007/s15010-024-02268-z (PMC11621180; doi:10.1007/s15010-024-02268-z)
Supplement: Supplementary file 1 — Supplementary file1 (DOCX 18 KB) [file 15010_2024_2268_MOESM1_ESM.docx]

**SUPPLEMENTARY MATERIAL**

**Supplementary Table S1. Checklist of items according to STROBE document*.***

|  | **Recommendation** | **Assessment in article** |
| --- | --- | --- |
| Title and abstract | (*a*) Indicate the study design with a commonly used term in the title or abstract | Study design specified in title and abstract |
|  | (*b*) Provide an informative and balanced summary in the abstract of what was done and what was found | Balanced summary included in the abstract |
| Background/ rationale | Explain the scientific background and rationale for the investigation being reported | The scientific background and rationale are included in the Introduction |
| Objectives | State specific objectives, including any pre-specified hypotheses | Pre-specified hypothesis and objectives are stated in the Introduction |
| Study design | Present key elements of study design early in the paper | Study design described in the first part of Methods |
| Setting | Describe the setting, locations, and relevant dates, including periods of recruitment, exposure, follow-up, and data collection | Described in Methods |
| Participants | (*a*) Give the eligibility criteria and the sources and methods of selection of participants. Describe methods of follow-up | Described in Methods |
|  | (*b*) For matched studies, give matching criteria and number of exposed and unexposed | Described in Methods |
| Variables | Clearly define all outcomes, exposures, predictors, potential confounders and effect modifiers. Give diagnostic criteria, if applicable | Defined in Methods |
| Data sources/ measurement | For each variable of interest, give sources of data and details of methods of assessment (measurement). Describe comparability of assessment methods if there is more than one group | Specified in Methods. The same data collection methods were used for both groups. |
| Bias | Describe any efforts to address potential sources of bias | Selection bias: inclusion of consecutive cases. Information bias: use of well defined, standard, easy to collect variables.  Use of hard outcome variables. |
| Study size | Explain how the study size was arrived at | The attempted sample size was specified in Methods |
| Quantitative variables | Explain how quantitative variables were handled in the analyses. If applicable, describe which groupings were chosen and why | Quantitative variables were handled as such. |
| Statistical methods | (*a*) Describe all statistical methods, including those used to control for confounding | Included in Methods |
|  | (*b*) Describe any methods used to examine subgroups and interactions | No subgroups analysed. |
|  | (*c*) Explain how missing data were addressed | Variables with >25% missing data were not accounted for multivariate analysis. |
|  | (*d*) If applicable, explain how loss to follow-up was addressed | Patients with missing data in the study outcomes were excluded due to loss to follow up. |
|  | (*e*) Describe any sensitivity analyses | Not applied |
| Participants | (a) Report numbers of individuals at each stage of study—eg numbers potentially eligible, examined for eligibility, confirmed eligible, included in the study, completing follow-up, and analysed | Included in Results and Figure 1 |
|  | (b) Give reasons for non-participation at each stage | Specified in Figure 1 |
|  | (c) Consider use of a flow diagram | Figure 1 |
| Descriptive data | (a) Give characteristics of study participants (eg demographic, clinical, social) and information on exposures and potential confounders | Table 1 |
|  | (b) Indicate number of participants with missing data for each variable of interest | Table 1 |
|  | (c) Summarise follow-up time (eg, average and total amount) | Information up to 90 days was available for all included patients |
| Outcome data | Report numbers of outcome events or summary measures over time | Included in Results |
| Main results | (*a*) Give unadjusted estimates and, if applicable, confounder-adjusted estimates and their precision (eg, 95% confidence interval). Make clear which confounders were adjusted for and why they were included | Specified in Results |
|  | (*b*) Report category boundaries when continuous variables were categorized | No category boundaries applied. |
|  | (*c*) If relevant, consider translating estimates of relative risk into absolute risk for a meaningful time period | Not applicable |
| Other analyses | Report other analyses done—eg analyses of subgroups and interactions, and sensitivity analyses | Specified in Methods and Results |
| Key results | Summarise key results with reference to study objectives | Specified in Abstract and Discussion |
| Limitations | Discuss limitations of the study, taking into account sources of potential bias or imprecision. Discuss both direction and magnitude of any potential bias | Included in Discussion |
| Interpretation | Give a cautious overall interpretation of results considering objectives, limitations, multiplicity of analyses, results from similar studies, and other relevant evidence | Included in Discussion |
| Generalisability | Discuss the generalisability (external validity) of the study results | Included in Discussion |
| Funding | Give the source of funding and the role of the funders for the present study and, if applicable, for the original study on which the present article is based | Included |
